# Epileptic Encephalopathy Due to *BRAT1* Pathogenic Variants

**DOI:** 10.15844/pedneurbriefs-30-12-1

**Published:** 2016-12

**Authors:** Siddharth Srivastava, Sakkubai Naidu

**Affiliations:** 1 Department of Neurology, Boston Children’s Hospital, Boston, MA; 2 Hugo W. Moser Research Institute at Kennedy Krieger Institute, Baltimore, MA

**Keywords:** Intractable Epilepsy, Microcephaly, Hypertonia, Apnea, *BRAT1*

## Abstract

Investigators from Institut für Medizinische Genetik und Humangenetik have highlighted the role of compound heterozygous *BRAT1* variants in two German brothers with variable presentations of intractable epilepsy, poor development, postnatal microcephaly, hypertonia, apnea, and infantile/childhood death.

Investigators from Institut für Medizinische Genetik und Humangenetik have highlighted the role of compound heterozygous *BRAT1* variants in two German brothers with variable presentations of intractable epilepsy, poor development, postnatal microcephaly, hypertonia, apnea, and infantile/childhood death. The older brother (Pt 1) died at 5.75 years, while the younger brother (Pt 2) died at 2 months. Seizure onset occurred at 5 months in Pt 1 and at birth in Pt 2 (and possibly in utero). Seizures were myoclonic, refractory to treatment, and accompanied by apnea, bradycardia (Pt 2), and focal/multifocal epileptiform discharges. Microcephaly was severe. Pt 1 achieved some turning and Pt 2 acquired no milestones. Appendicular hypertonia was present in both. Pt 2’s brain MRI was normal; Pt 1’s brain MRI showed corpus callosum thinning, enlarged CSF fluid spaces, and delayed myelination. Next-generation sequencing (NGS) of the disease-associated genome (~2800 genes) revealed a compound heterozygous variant in *BRAT1* [c.638_639insA (p.V214fs189*); c.1134+1G>A], confirmed in both siblings. The frameshift variant, which was maternally inherited, is a known change associated with lethal neonatal rigidity and multifocal seizure syndrome (RMFSL). The other variant, which was paternally inherited, alters splicing, evident by reduced *BRAT1* mRNA expression in the father. Skeletal muscle biopsy from Pt 2 revealed myofiber immaturity, decreased cyclooxygenase staining, and decreased cytochrome c oxidase activity. [1]

COMMENTARY. This study expands the knowledge surrounding *BRAT1*-related disorders, particularly its clinical heterogeneity. Some of the first reports of this disorder characterized it as a particularly severe, rapidly progressive, intractable epileptic encephalopathy with age of presentation at birth or shortly thereafter [2, 3]. While these earlier investigations suggested it is lethal in the first few months of life, this present report points to increased survival into childhood (Pt 1) as one of the features of the disorder. Moreover, other manifestations in Pt 1 – later onset of epilepsy, postnatal microcephaly, and hypertonia – suggest a less affected phenotype. In fact, in addition to the severe lethal form known as RMFSL, both mild and moderate forms of *BRAT1*-related disorders may exist. Mildly affected individuals may present with intellectual disability without epilepsy/seizures, ataxia, cerebellar atrophy, and continued survival through late childhood [4]. Given the phenotypic differences seen with siblings, intrafamilial variability can occur.

This study also demonstrates that mitochondrial dysfunction may be a hallmark of *BRAT1*-related disorders. Pt 2’s skeletal muscle biopsy showed evidence of impaired mitochondrial energy production. In another study, BRAT1 knockdown resulted in cells with increased glucose requirements, increased reactive oxygen species levels, and decreased ATP production [5]. Defects in mitochondrial metabolism, combined with defects in some of the other roles of BRAT1 including DNA repair and cell growth [6], may account for some of the presentations of this disorder.

Finally, this study highlights the role of NGS in diagnosing causes of epileptic encephalopathy. Depending on the laboratory, *BRAT1* may not be one of the genes sequenced as part of an epileptic encephalopathy panel. Increased awareness of this disorder, combined with utilization of NGS, may lead to earlier diagnoses.
